# Confirmation of the shell-boring oyster parasite *Polydora websteri* (Polychaeta: Spionidae) in Washington State, USA

**DOI:** 10.1038/s41598-020-60805-w

**Published:** 2020-03-03

**Authors:** Julieta C. Martinelli, Heather M. Lopes, Lorenz Hauser, Isadora Jimenez-Hidalgo, Teri L. King, Jacqueline L. Padilla-Gamiño, Paul Rawson, Laura H. Spencer, Jason D. Williams, Chelsea L. Wood

**Affiliations:** 1 School of Aquatic and Fishery Sciences, University of Washington, Seattle, WA 98105 USA; 2 Washington Sea Grant, University of Washington, Shelton, WA 98584 USA; 3 School of Marine Sciences, University of Maine, Orono, ME 04469 USA; 4 Department of Biology, Hofstra University, Hempstead, NY 11549 USA

**Keywords:** Ecological epidemiology, Invasive species

## Abstract

Invasions by shell-boring polychaetes such as *Polydora websteri* Hartman have resulted in the collapse of oyster aquaculture industries in Australia, New Zealand, and Hawaii. These worms burrow into bivalve shells, creating unsightly mud blisters that are unappealing to consumers and, when nicked during shucking, release mud and detritus that can foul oyster meats. Recent findings of mud blisters on the shells of Pacific oysters (*Crassostrea gigas* Thunberg) in Washington State suggest a new spionid polychaete outbreak. To determine the identity of the polychaete causing these blisters, we obtained Pacific oysters from two locations in Puget Sound and examined them for blisters and burrows caused by polychaete worms. Specimens were also obtained from eastern oysters (*Crassostrea virginica* Gmelin) collected in New York for morphological and molecular comparison. We compared polychaete morphology to original descriptions, extracted DNA and sequenced mitochondrial (cytochrome c oxidase I [mtCOI]) and nuclear (small subunit 18S rRNA [18S rRNA]) genes to determine a species-level molecular identification for these worms. Our data show that *Polydora websteri* are present in the mud blisters from oysters grown in Puget Sound, constituting the first confirmed record of this species in Washington State. The presence of this notorious invader could threaten the sustainability of oyster aquaculture in Washington, which currently produces more farmed bivalves than any other US state.

## Introduction

The most notorious invasive species simultaneously compromise the function of ecosystems and jeopardize the human societies that depend upon those ecosystems. Among these invaders is the spionid polychaete *Polydora websteri* Hartman, commonly known as a “mud worm” or “mud blister worm”^1^, which bores into the shells of molluscs^2^. By creating unsightly blisters on the shells of their commercially important hosts, these pests have led to significant economic losses for shellfish aquaculture^3^. *Polydora websteri* can infest a variety of mollusc hosts (see reviews^4–6^), including oysters^7–10^, mussels^11–13^, scallops^14–16^, and abalone^17^.

*Polydora websteri* and related polydorins (*sensu*^18^; a group of nine spionid genera with a modified fifth chaetiger) have compromised and collapsed oyster aquaculture industries around the world. In the late 1800s, the introduction of *Polydora websteri* with translocated oysters caused subtidal oyster beds in New South Wales, Australia to disappear^8,19–22^. When oyster transplants from Kaneohe Bay brought *Polydora websteri* to Kakuku, Hawaii, the introduction caused extensive damage to shellfish production^23,24^. Oyster farms on the east coast of the United States have been plagued with *Polydora websteri* infestations since the 1940s, resulting in substantial oyster farm losses^25–27^. In addition, high mortalities of the Japanese scallop *Mizuhopecten yessoensis* (Jay 1857) in British Columbia, Canada were attributed to *Polydora websteri*^28^. These examples attest to the ability of *Polydora websteri* to successfully invade new locations and, once established, to significantly impact aquaculture production.

*Polydora websteri* infestations are detrimental to oyster aquaculture because the worms result in unsightly blisters on oyster valves, decreasing market value. The mud worm typically has a pelagic larval stage, after which the larvae settle onto the external side of a calcareous shell^22,29,30^. The worm then forms a U-shaped burrow with two exterior openings^27,31^. As they grow, burrows breach the inner surface of the valve, causing the host to produce a brittle layer of nacre that walls off the burrow^4,10,32–34^. The worm continues to expand this burrow beneath the thin, calcareous layer produced by its host; as this space fills with detritus, mud, and worm feces, a “mud blister” is formed^33,35^. Blisters can be irregular in shape and darkly colored, compromising the presentation of oysters served on the half-shell (only the cupped or left valve is used for serving the oyster)^36^. Moreover, if a blister is nicked during oyster shucking, the mud and feces will foul the oyster meat, rendering it inedible^3^. This is particularly problematic for oyster-growing areas where a large proportion of production goes to the half-shell market.

In addition to their detrimental impact on aquaculture production, heavy mud worm infestations can also impact shell integrity, growth, and survivorship of mollusc hosts^37^. When infested with *Polydora ciliata* Johnston, the gastropod *Littorina littorea* (Linnaeus) has significantly reduced shell strength relative to uninfested individuals, making the infested gastropods more vulnerable to predation^38^. Pacific oysters (*Crassostrea gigas*) infested by the polydorids *Polydora hoplura* Claparède, *Polydora cornuta* Bosc, and *Boccardia semibranchiata* Radashevsky grow more slowly and have poorer body condition than do uninfested oysters^39^. Glycogen, protein, and lipid content relative to the shell cavity volume are lower in infested compared to uninfested *Crassostrea* sp oysters^7,25,40^. Additionally, polydorins have been shown to increase mortality rates in Pacific oysters that are heavily infested^41,42^. These negative effects on growth and survivorship may be caused by the energetic demands of worm-induced nacre production^7,40,43–46^; that is, infested hosts may need to invest energy into isolating their tissue from the worm by building multiple costly shell layers instead of investing that energy into their own growth and reproduction^47^. Given these impacts on the growth and reproduction of the host, *Polydora websteri* outbreaks may affect more than just the bottom line of the shellfish industry; they may also compromise the important ecosystem services provided by filter-feeding shellfish species^48^.

*Polydora websteri* has been reported from locations all over the world (see reviews^10,49–52^), but due to its complex taxonomic history (see^53–55^), many records remain to be confirmed. Some historical reports of *Polydora ciliata* (a non-boring species) have been re-identified as other, shell-boring polydorin species, including *Polydora websteri*^9^, and additional erroneous historical reports might exist. *Polydora websteri* is believed to be of Asian origin, and genetic homogeneity among North American, Hawaiian, and Asian specimens suggests that human-mediated transport produces high levels of connectivity among populations^10^. Although *Polydora websteri* has been predicted to be present in Washington, USA^56^ based on records of its presence to the north in British Columbia^28,57^, and to the south in Oregon and California (e.g.^29,58–60^), it has never before been described from Washington. Its potential absence is a fortunate circumstance; as the United States’ leading producer of bivalve shellfish, Washington State’s bivalve aquaculture brings in over $92 million dollars in revenue annually^61^. Of Washington State’s cultured shellfish production, Pacific oysters (*Crassostrea gigas*) contribute 38% by weight and 38% by value^61^. Pacific oysters are also culturally important to local communities, Native American tribes, family-owned farms, and recreational farmers and collectors^62^. As the industry has evolved in recent years, producers have shifted to the lucrative half-shell market, where the shell is presented to the consumer^61^. Utilizing the half-shell market, Washington’s oyster industry is structured in such a way that a *Polydora websteri* outbreak could cause extensive damage if infested oysters result in lost value.

**Figure 1:**
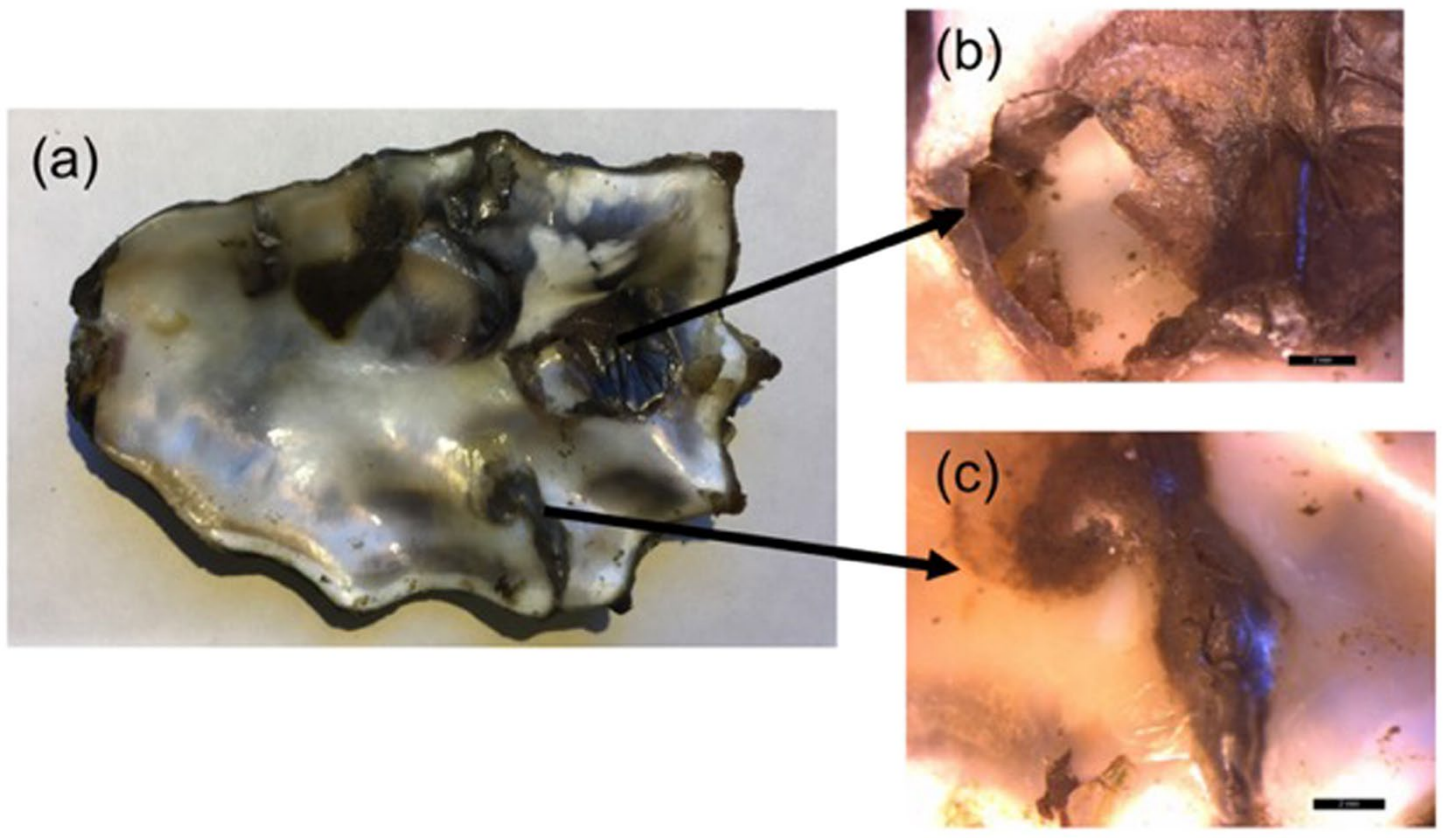
*Crassostrea gigas* infested with *Polydora websteri* collected from Oakland Bay, WA. Pathology shown is associated with shell-boring mud worms. (**a**) Inner surface of an infested valve, (**b**) opened mud blister, and (**c**) closed mud blister filled with mud, detritus, and worm feces. In (**b**,**c**), scale bar indicates 2 mm.

**Figure 2:**
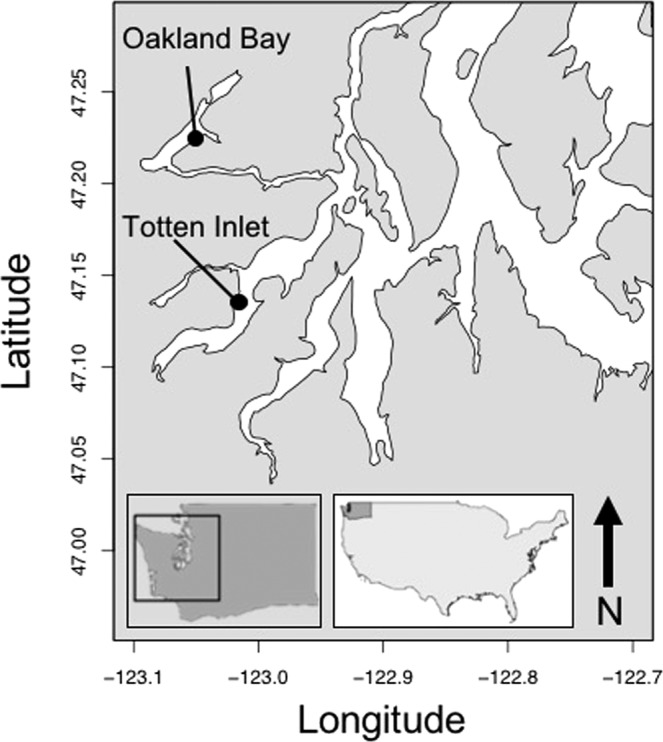
Map of sampling sites in Southern Puget Sound, Washington State. Inset images show the position of Puget Sound in Washington State, and the position of Washington State in the United States. Oysters were obtained from culture sites in Oakland Bay (n = 69) and Totten Inlet (n = 114).

Washington State oysters have long been prized for the consistent color of their inner valves, in contrast to the mud-blister-blemished valves of oysters grown in other parts of North America (T. King, *personal communication*). However, in recent years, one of us (TK) began noticing mud blisters on the valves of Pacific oysters (*Crassostrea gigas*) grown in Puget Sound (Fig. 1). Site visits with local oyster growers confirmed these observations, and suggested that Washington State – a globally important aquaculture region^61^ – may be experiencing a *Polydora websteri* outbreak. To confirm the species identity of the organisms causing these blisters, we sampled Pacific oysters from two bays in the Puget Sound (Fig. 2), an estuary in Washington State with extensive commercial oyster beds. In addition, specimens of *Polydora websteri* in eastern oysters (*Crassostrea virginica*) from Long Island, New York (<65 km across the Long Island Sound from the type locality in Milford, CT), were collected for morphological and molecular comparisons. Worms were recovered from shell blisters and burrows, and identified to species using morphological traits, as well as mitochondrial cytochrome c oxidase subunit I [mtCOI] and nuclear small subunit 18S rRNA [18S rRNA] gene sequences. Our results constitute the first formal report of a shell-boring polychaete from Puget Sound, and the first report of the notorious pest *Polydora websteri* in Washington State.

## Results

### Morphological identification

**Figure 3:**
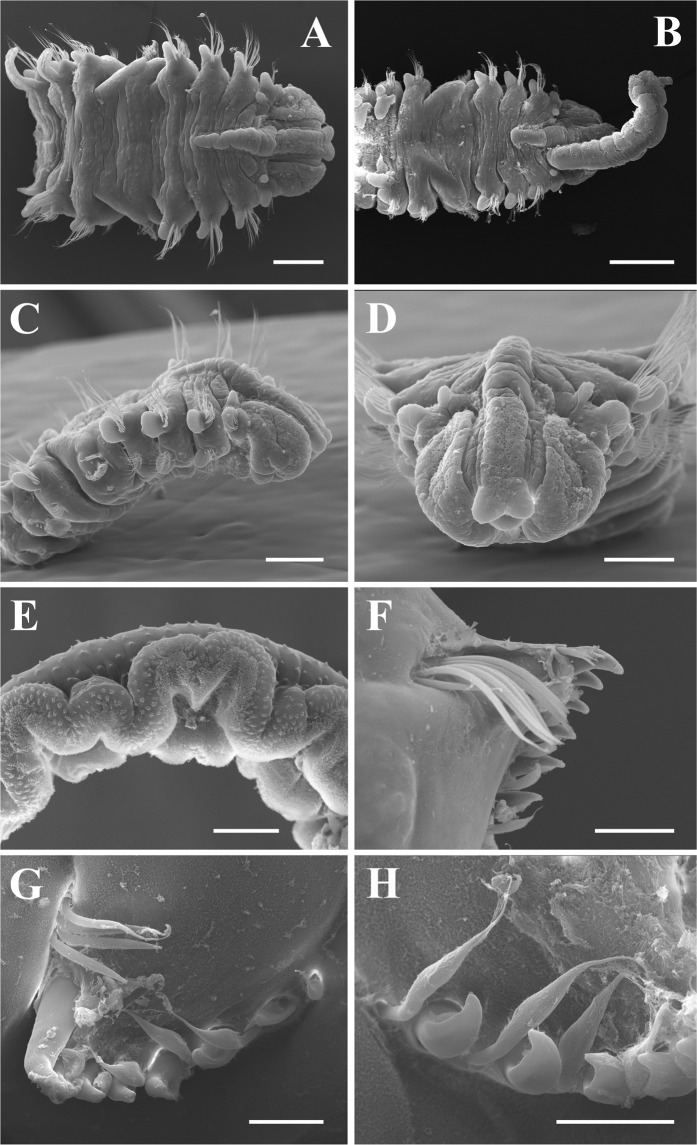
*Polydora websteri* from Oakland Bay, Washington extracted from *Crassostrea gigas*. (**A**) Anterior dorsal view of specimen lacking palps (USNM 1606131). (**B**) Anterior dorsal view of specimen with basal portion of right palp attached (USNM 1606127). (**C**) Anterior, right lateral view, same specimen as in A (USNM 1606131). (**D**) *En face* view of specimen showing anterior end of prostomium, same specimen as in A (USNM 1606131). (**E**) Lateral view of middle portion of palp, palp removed from specimen shown in B (USNM 1606131). (**F**) Dorsal view of fifth chaetiger spines (USNM 1606126). (**G**) Dorsal view of fifth chaetiger spines, same specimen as in B (USNM 1606127). (**H**) Lateral view of fifth chaetiger spines, close-up, same specimen as in B (USNM 1606127). Scale bars A–C = 250 µm, D = 200 µm, E = 100 µm, F = 50 µm, G, H = 25 µm.

**Figure 4:**
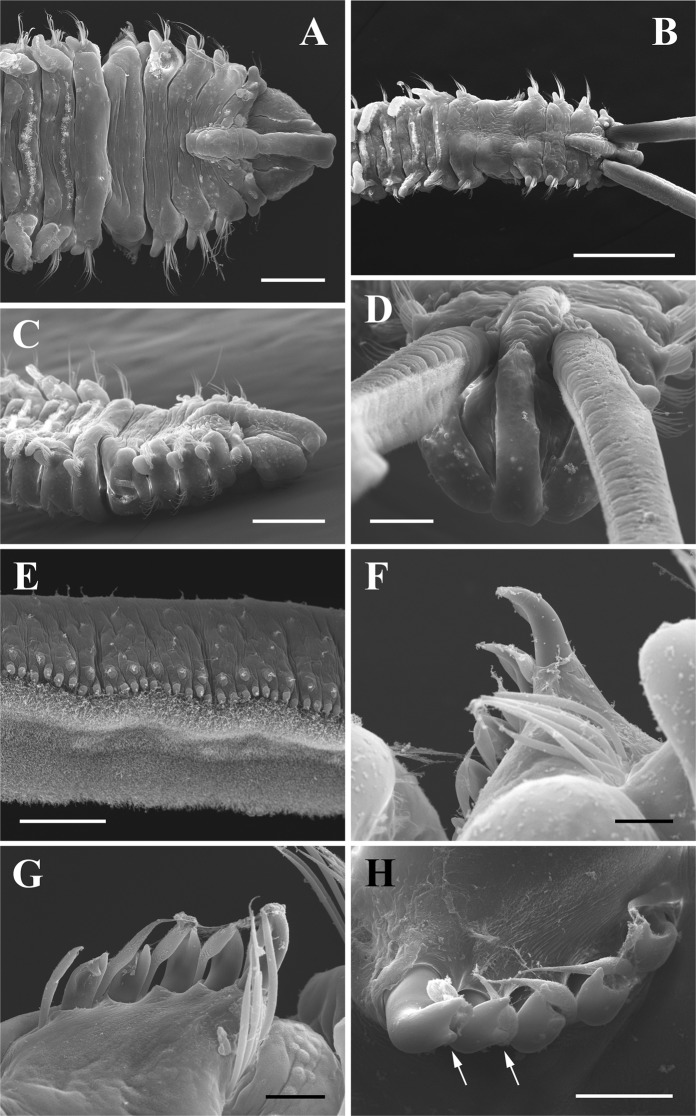
*Polydora websteri* from Long Island, New York extracted from *Crassostrea virginica*. (**A**) Anterior dorsal view of specimen lacking palps (USNM 1606133). (**B**) Anterior dorsal view of specimen with palps (USNM 1606134). (**C**) Anterior, right lateral view, same specimen as in A (USNM 1606133). (**D**) *En face* view of specimen showing anterior end of prostomium, same specimen as in B (USNM 1606134). (**E**) Lateral view of middle portion of palp, same specimen as in B (USNM 1606134). (**F**) Dorsal view of fifth chaetiger spines (USNM 1606135). (**G**) Dorsal view of fifth chaetiger spines, same specimen as in B (USNM 1606134). (**H**) Lateral view of fifth chaetiger spines, close-up, arrows indicate subdistal “tooth,” same specimen as in A (USNM 1606133). Scale bars A = 200 µm, B = 500 µm, C = 250 µm, D = 100 µm, E = 50 µm, F–H = 25 µm.

Specimens from both Washington (Fig. 3) and New York (Fig. 4) matched the taxonomically important features of *Polydora websteri* in the original description^27^, redescription^54^ and more recent reports^9,10,52,63^.

### *Polydora websteri* Hartman in Loosanoff and Engle, 1943

#### Morphology of adults

Complete specimens of up 60–100+ chaetigers, approximately 1.0-mm wide at chaetiger 7. Prostomium bilobed anteriorly (Figs. 3A and 4A,C), sometimes appearing weakly notched but specimens must be examined *en face* (Figs. 3D and 4D) or ventrally to determine the bilobed nature. Caruncle extending posteriorly to end of chaetiger 2 (Figs. 3B and 4A,B) or chaetiger 3 (Fig. 3A); small, round, black eyes variable in number (0–4), commonly 4 present in trapezoidal pattern between palps; occipital tentacle always absent (Figs. 3A–D and 4A–D). Palps extending posteriorly for approximately 10–15 chaetigers, palps with a ventral food groove lined by frontal cilia, non-motile cirri on papillae along lateral edges of food groove and scattered on the abfrontal surface (Figs. 3E and 4E). When contracted, palps exhibit scalloped lateral edge (Fig. 3E); when relaxed, palps exhibit straight lateral edge (Fig. 4E). Longitudinal black pigment band was observed along lateral edge of palp, in some specimens pigmentation superficially resembled transverse bands when the palps were contracted. Color of body in alcohol opaque white to light tan. Methyl green staining (NY specimen) occurred on ventral sides of chaetigers 1–4, along the sides of the caruncle (midway and between the palps), and as granular patches on dorsal surface near base of branchiae from middle segments posteriorly.

Chaetiger 1 with neurochaetae, without notochaetae, with digitiform notopodial lobes (Figs. 3A–D and 4A–D). Cilia of lateral organs present between notopodial lobe and neuropodial lobes of chaetiger 1 and present between notopodial and neurochaetae of chaetiger 2 (additional lateral organs may be present on more posterior chaetigers but have been lost during fixation). Winged capillary notochaetae of chaetigers 2–4, 6 and subsequent chaetigers arranged in three successive rows, reduced to thin notochaetae in posterior chaetigers; no specialized posterior notochaetae. Winged capillary neurochaetae of chaetigers 2–4, 6 and subsequent chaetigers arranged in two vertical rows; 5–8 bidentate hooded hooks begin on chaetiger 7, not accompanied by capillaries, increasing to 8–10 in series at chaetiger 9; hooks with approximately right angle between main fang and shaft, with constriction on shaft; glandular pouches near base of ventral-most hooded hook in chaetigers 7–8, observed by the external portion of secretory cells which appear as small papillae.

Chaetiger 5 almost twice as large as chaetigers 4 and 6, with slightly curved row of 5–7 exposed major spines and additional embedded spines, major spines alternating with pennoned companion chaetae, sometimes exhibiting frayed tips; anterior dorsal fascicle of 4–6 geniculate notochaetae present and tips directed posteriorly, ventral fascicle of 4–6 winged capillary neurochaetae below row of major spines (Figs. 3F–H and 4F–H). Major spines falcate, with shallow lateral flange, most visible in younger, posterior spines (Figs. 3G,H and 4G,H); older, anterior spines may appear to have lateral tooth but this is the remains of the worn flange (Fig. 4H).

Branchiae from chaetiger 7 (Figs. 3A,B and 4A–C), free from notopodial postchaetal lamellae, reaching full size at chaetigers 9–10 and overlapping middorsally, diminishing in length posteriorly and absent from posteriormost chaetigers; ciliary bands present on dorsal surface of chaetigers between branchiae (Figs. 3A and 4A,B). Pygidium broad, cup-shaped with dorsal gap.

#### Remarks

**Figure 5:**
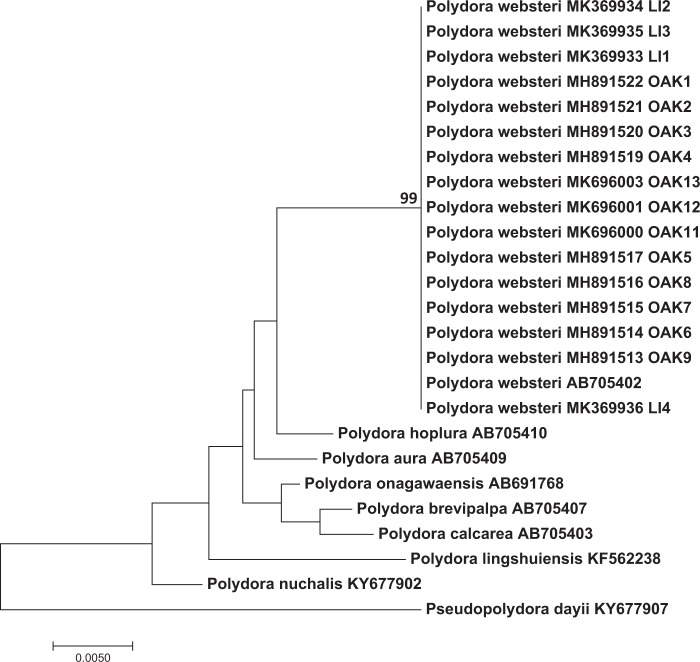
Neighbor-joining phylogeny based on Kimura 2-parameter distances using trimmed 18S1 rRNA sequences (1000 replicates). The optimal tree with the sum of branch length = 0.087 is shown. Clades which were recovered in greater than 80% of replicate trees in the bootstrap test are shown along the branches leading to the clade nodes. The tree is drawn to scale, with branch lengths in the same units as those of the evolutionary distances used to infer the phylogenetic tree. The rate variation among sites was modeled with a gamma distribution (shape parameter = 1). *Pseudopolydora dayii* (KY677907) was used as an outgroup. New sequences reported in this study labeled with OAK and LI were collected in Oakland Bay and Long Island respectively.

The specimens of *Polydora websteri* from WA and NY match the taxonomically important features of those in the original description (Hartman in^27^), redescription^54^, and more recent reports^9,10,52,63^. Although the caruncle was described as extending to end of chaetiger 2 in the lectotypes of *Polydora websteri*^54^, others have found it reaching mid-chaetiger 3^9^, end of chaetiger 3^63^ or to chaetiger 4^10^. In the present specimens the caruncle extended to mid-chaetiger 2 in some and through end of chaetiger 3 in others. As noted by^54^, in lateral view the caruncle can appear to extend further posteriorly because the middorsal boundaries between chaetigers are displaced backward in comparison to lateral boundaries (e.g., Fig. 4A caruncle extends to posterior end of chaetiger 2 but in lateral view it appears to extend to mid-chaetiger 3). The palps of this species may have a black line of pigmentation along the lateral edge of palp, as shown by others (see Fig. 1 in^9^; Fig. 5D in^63^. However, when the palps are contracted (Fig. 3E) this pigmentation can become concentrated and appear as dark bands (as noted by other researchers for this species: Fig. 1a in^10,52^). After fixation in formalin and preservation in ethanol, the differences in palp pigmentation patterns are retained (e.g., USNM 1606136 from NY with line of pigmentation; USNM 1606128 from WA with bands of pigmentation). The methyl green staining pattern is similar to that observed by Read^9^, although he noted granular staining in anterior branchiae. Major spines are falcate, with a shallow lateral flange (Figs. 3G,H and 4G,H); although older anterior spines may appear to have a lateral tooth, this is the remains of the worn flange (Fig. 4H). Lateral organs (=lateral ciliated organs; see^64^) were present on chaetigers 1 and 2, but presence/absence on posterior chaetigers should be confirmed based on specimens fixed in glutaraldehyde. One of the specimens from WA (USNM 1606127; Fig. 3B) had hooded hooks beginning on chaetiger 6, but this seems to be an abnormal specimen; all other reports and specimens examined herein show that the hooded hooks begin on chaetiger 7.

#### Prevalence

Of the 183 oysters collected from south Puget Sound, 40% (74 individuals) were infested with at least one blister or burrow. Among oysters from Oakland Bay, in South Puget Sound (Fig. 2), 53% were infested; among oysters from Totten Inlet, 34% were infested.

#### Molecular identification

**Figure 6:**
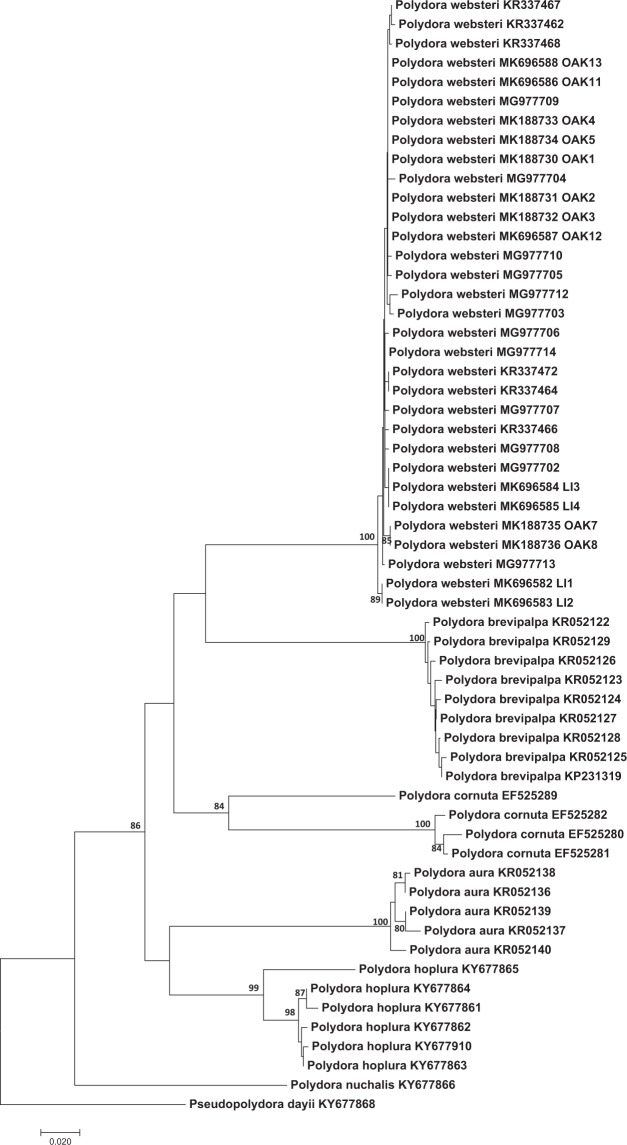
Neighbor-joining phylogeny based on Kimura 2-parameter distances using trimmed mtCO1 sequences (1000 replicates). The optimal tree with the sum of branch length = 1.20 is shown. Clades which were recovered in greater than 80% of replicate trees in the bootstrap test are shown along the branches leading to the clade nodes. The tree is drawn to scale, with branch lengths in the same units as those of the evolutionary distances used to infer the phylogenetic tree. The rate variation among sites was modeled with a gamma distribution (shape parameter = 1). *Pseudopolydora dayii* (KY677907) was used as an outgroup. New sequences reported in this study labeled with OAK and LI were collected in Oakland Bay and Long Island respectively.

All of the specimens identified as *Polydora websteri* by morphological analysis were confirmed as belonging to that species by molecular analyses. Of the 13 specimens collected from Oakland Bay sequenced at 18S rRNA, 12 were identified as *Polydora websteri* (Table 1). Our 18S rRNA neighbor-joining phylogeny indicated that these 12 sequences clustered in the same clade as the *Polydora websteri* sequences from Genbank. As Rice *et al.*^10^ reported for sequences of *Polydora websteri* from several Atlantic coast, Gulf coast, and Hawaiin specimens, all *Polydora websteri* 18S rRNA sequences in our study were identical. The Oakland 18S rRNA sequences also match the four sequences from Long Island (Fig. 5) with the exception of sequence LI4B which had several unresolved bases. There was more structure evident in the phylogeny based on sequences from the mtCO1 gene. Even so, ten  worms from Oakland Bay and the four from Long Island that were sequenced with mtCOI also clustered with *Polydora websteri* in the mtCOI neighbor-joining phylogeny (Fig. 6, Table 1) and were clearly divergent from all other published mtCO1 sequences for *Polydora sp* on Genbank. Some worms collected from Oakland Bay and all Totten Inlet worms were not included in our molecular analyses as we do not have clear morphological identifications and there are not matching, published molecular data available on Genbank for these specimens. In summary, however, both 18S rRNA and mtCOI sequence analysis indicates that *Polydora websteri* is present in Oakland Bay, Puget Sound, Washington.

**Table 1:** Taxa, sampling location data, museum catalog numbers of voucher specimens and GenBank accession numbers of specimens for which we have matching molecular and morphological identifications.

Molecular ID	Morphological ID	Worm ID on trees	Location and host	Coords.	Date	USNM Voucher Number (SEM or EtOH)	GenBank Accession Numbers	
							*18S*	*COI*
*Polydora websteri*	*Polydora websteri*	—	Oakland Bay, Washington State, USA; from shells of *Crassostrea gigas*	47°13′ 45.93″, –123°3′ 19.43″	15 Aug 2018	1606126 (SEM)	MK695999	—
*Polydora websteri*	*Polydora websteri*	—				1606127 (SEM)	—	—
*Polydora websteri*	*Polydora websteri*	—				1606128 (EtOH)	MK696002	—
*Polydora websteri*	*Polydora websteri*	OAK11				1606129 (SEM)	MK696000	MK696586
*Polydora websteri*	*Polydora websteri*	OAK12				1606130 (SEM)	MK696001	MK696587
*Polydora websteri*	*Polydora websteri*	OAK13				1606131 (SEM)	MK696003	MK696588
*Polydora websteri*	*Polydora websteri*	—				1606132 (SEM)	—	—
*Polydora websteri*	*Polydora websteri*	LI1	North Sea Harbor, Long Island, New York, USA; from shells of *Crassostrea virginica*	40°56′ 24.13″N, 72°25′ 3.97″W	12 Sep 2018	1606133 (SEM)	MK369933	MK696582
*Polydora websteri*	*Polydora websteri*	LI2	Same as above	Same as above		1606134 (SEM)	MK369934	MK696583
*Polydora websteri*	*Polydora websteri*	LI3	Same as above			1606135 (SEM)	MK369935	MK696584
*Polydora websteri*	*Polydora websteri*	LI 4	Same as above			1606136 (EtOH)	MK369936	MK696585

The rest of the specimens that only have molecular identification are presented in Supplementary Table 1. USNM = National Museum of Natural History, Smithsonian Institution, Washington D.C., USA; SEM = specimen prepared for scanning electron micrograph; EtOH = specimen preserved in ethanol. Specimens that were unresolved in the phylogenetic trees are not included in this table.

Haplotype diversity, nucleotide diversity and the average number of nucleotide differences were all substantially lower for the 18S rRNA gene sequences relative to the mtCO1 gene sequences (Table 2) as would be expected for the more conserved nuclear locus. For mtCOI, the mean intraspecific for *Polydora websteri* was 0.002 (n = 21, Table 3) and the interspecific distances between *Polydora websteri* and the other species ranged between 0.185 and 0.240 (Table 3). For nuclear 18S, the mean intraspecific distance for *Polydora websteri* was 0.00 (n = 21, Table 4) and the interspecific distances between *Polydora websteri* and the other species was 0.02 (Table 4).

**Table 2:** Sequences alignment statistics for 18S and COI sequences for *Polydora websteri*.

Variable	18S	COI
Sample size	16	14
Final length of aligned sequences in bp	614	554
No. variable nucleotides	22/614	164/554
Haplotype diversity	0.009	0.135
Nucleotide diversity	0.01	0.03
Transitions/transversions ratio	1.02	1.31

Values were calculated using MEGA7.0.26.

**Table 3:** Intraspecific and interspecific Kimura-2-parameter (KP2) distances for the mitochondrial COI sequences of the *Polydora websteri* from our dataset and GenBank.

Species	No. indiv.	Origin	GenBank accession numbers	Intrasp. distance	Interspecific distance					
					1	2	3	4	5	6
1. *Polydora aura*	5	China	KR052136 - 40	0.0124						
2. *Polydora brevipalpa*	11	China	KP231319 - 25, KR052121 - 4	0.0026	0.258					
3. *Polydora hoplura*	10	South Africa	KY677860 - 3, KY002977 - 82	0.0202	0.176	0.190				
4. *Polydora lingshuiensis*	8	China	KU525630 - 37	0.0051	0.222	0.207	0.202			
5. *Polydora nuchalis*	4	South Africa	KY677866, KY002983-85	0	0.215	0.267	0.210	0.245		
6. *Polydora websteri*	21	South Africa, China, United States	KR337469 - 72, KY002986 - 88, samples from this contribution	0.002	0.240	0.185	0.208	0.218	0.261

**Table 4:** Intraspecific and interspecific Kimura-2-parameter (KP2) distances for the nuclear 18S sequences of *Polydora websteri* from our dataset and GenBank.

Species	No. indiv.	Origin	GenBank accession numbers	Intrasp. distance	Interspecific distance		
					1	2	3
1. *Polydora brevipalpa*	8	China	KP231289 - 96	0.00			
2. *Polydora lingshuiensis*	5	China	KF562236 - 40	0.00	0.02		
3. *Polydora websteri*	21	South Africa, Japan, China, Australia, United Sates	KY003046 - 48, KY677904 - 06, AB705402, AB705405, KP231302	0.00	0.02	0.02	

## Discussion

Our findings constitute the first report of *Polydora websteri* in Washington State, United States. The presence of this shell-boring polydorin poses a danger to the region’s valuable oyster aquaculture industry. All worms from Oakland Bay that were identified as *Polydora websteri* based on diagnostic morphological features also clustered with GenBank sequences of *Polydora websteri* both in the 18S rRNA and mtCOI phylogenetic trees (Table 1, Figs. 5 and 6). Based on detailed morphological analysis, specimens of *Polydora websteri* from Oakland Bay (Fig. 3) matched previous descriptions and the newly collected material from Long Island, NY near the type locality (Fig. 4); the same specimens that we morphologically identified were also sequenced, and morphological and molecular diagnoses agreed. We therefore confirm the presence of *Polydora websteri*, a shell-boring mud worm, in the shells of Washington State Pacific oysters. *Polydora websteri* has never before been reported from Washington. This blister-forming species could endanger an aquaculture industry that provides both multi-million-dollar revenues ($92 million in 2015) and valuable ecosystem services to Washington State^61^.

The fact that *Polydora websteri* has never before been documented in Washington State oysters suggests a recent introduction, but it is also possible that the species has been present in the region for some time and has undergone a recent increase in prevalence perhaps associated to the aquaculture industry or environmental changes. Extensive exchange of shell and live oysters among regions in Washington continues to the present day, and to such an extent that *Polydora websteri* populations are genetically homogenous across broad swathes of their contemporary range^10^. Washington State has a long history of exchange with other oyster-growing regions^65^ and polydorin pelagic larvae may also have been introduced through ballast water^66,67^. Although it is likely that *Polydora websteri* is native to Asia and exotic to North America^10^, we suggest that *Polydora websteri* be considered cryptogenic in Washington State^68^ until further research can resolve its origins. Considering the species is distributed north and south of Washington (e.g.^28,29,57–60^), it is likely that the species has been present in this region but has never before been reported because it occurred only at low prevalence until recently. The prevalence of *Polydora websteri* is sensitive to environmental change. For example, increasing siltation can increase the susceptibility of *Crassostrea virginica* to *Polydora websteri*^69^. In contrast, reducing pH actually decreases susceptibility to infestation^70^. Because *Polydora websteri* can recruit to both live and dead oyster shells^30^, the expansion of the oyster aquaculture industry, oyster restoration, and increased density of oysters in beds across the state might have promoted an increase in transmission and prevalence if the polychaete was already present. Whatever their origin or how affected they are by changing conditions, the blister-forming polychaetes we document here are a new challenge for Washington State oyster growers and the government agencies charged with management of shellfish stocks.

Because *Polydora websteri* is a generalist pest^9,32,33^, it may impact other shellfish species of ecological, economic, and cultural importance to Washington State. An important example is the Olympia oyster (*Ostrea lurida)*, an overexploited native species that is the focus of intensive restoration efforts^71^. Mussels^11–13^, scallops^14–16^, and abalone (^17^see review in^4^) are also at risk. Given the important ecosystem services provided by filter-feeding shellfish species^48^, a polydorin outbreak could affect more than just the bottom line of the shellfish industry; ecosystem functioning is also at risk.

We were not able to definitively identify the majority of worms collected from Totten Inlet (Fig. 2) using our combined morphological and molecular approach. However, our work indicates that the Puget Sound region hosts several cryptogenic spionid polychaete species, all of which may pose a danger to the regions oyster aquaculture industry. In our research, we positively identified the notorious shell-boring polydorin, *Polydora websteri*, in commercially farmed Pacific oysters, providing the first formal documentation of this globally distributed pest in Washington State. The pathology caused by shell-boring mud worms results in unsightly blisters that reduce the market value of infested oysters, especially those served on the half-shell. Washington’s Pacific oyster industry is dominated by the half-shell market^61^, and given the high prevalence of infestation found in this study, these pests have the potential to threaten the valuable Pacific oyster aquaculture operations in Washington State.

## Methods

### Oyster collections

To assess whether shell-boring polychaetes were present in Washington Pacific oysters (*Crassostrea gigas*) and to confirm the species identity of these worms, we purchased 183 commercially grown oysters from retail shellfish farms in Washington State, USA. Of these, 69 individuals came from Oakland Bay (47°13′45.93″, −123°3′19.43″, Fig. 2, Table 1), and 114 individuals were from Totten Inlet (47°9′43.09″, −122°59′19.62″, Fig. 2, Table 1). Both sites are in South Puget Sound, a region that yields 37% of the total mass and 58% of the value of shellfish produced annually by Washington State^61^. For comparison, we also collected commercially-grown eastern oysters (*Crassostrea virginica* Gmelin) from North Sea Harbor, Long Island, New York, USA (40°56′24.13″N, 72°25′3.97″W, Table 1), less than 65 kilometers from the type locality of *Polydora websteri* (“mouth of the Milford River”^27^, presumably near the mouth of the Wepawaug River that flows into the Milford Harbor, CT).

### Worm collections

All oysters were shucked, and the soft tissues removed. We observed right and left valves under a stereomicroscope for indications of mud worm infestation, such as burrows and blisters. All oysters (with or without infestation) were photographed and measured (height and length of the shell) using a digital caliper (results in Supplementary Table 2). We removed any worms present in blisters or burrows with a probe or forceps, or by fracturing shells with a hammer to expose worms in their burrows. Once removed from the shell, we photographed the worms and fixed them whole in 95% ethanol for molecular analysis or, in some cases, sectioned worms such that molecular analysis of a worm (typically middle and posterior chaetigers) could be linked with morphological analysis of the same worm (typically anterior ends).

### Morphological examination

For morphological examination, worms were fixed in 4% formalin/seawater overnight, washed in warm tap water, and transferred to 70% ethyl alcohol (EtOH) for storage. For examination with a scanning electron microscope (SEM), the specimens were dehydrated in an ascending ethanol series through 100% EtOH. Drying was accomplished with a Samdri 795 Critical Point Dryer. Once dried, the specimens were mounted on aluminum stubs, coated with gold using an EMS-550 Sputter coater, and viewed with a FEI Quanta 250 SEM. Voucher specimens (Table 1) were deposited in the National Museum of Natural History, Smithsonian Institution, Washington DC, USA (USNM).

### Infestation prevalence

We considered any oyster that had at least one blister or burrow to be infested. Prevalence was calculated as the proportion of infested oysters in each sample. We also calculated the number of blisters/burrows per oyster.

### DNA extraction, PCR amplification, and sequencing

Within the family Spionidae, species display variable morphology, making it challenging to obtain an accurate species-level identification based solely on morphological traits^63,64,72^. For this reason, we combined the morphological analysis, described above, with sequencing and phylogenetic analysis of variation at the nuclear 18S rRNA and mitochondrial cytochrome c oxidase I [mtCOI] genes^73^. We followed the protocol of^73^ in using a molecular approach to identify worms recovered from blisters and burrows.

For a subset (n = 27) of the total number of worms vouchered (n = 107) and for four additional worms collected from Long Island, New York, we extracted DNA using DNeasy 96 Blood & Tissue Kit (Qiagen, Valencia, CA) following the manufacturers’ instructions. We used two genes for molecular identification: the nuclear 18S rRNA [18S rRNA] and the mitochondrial cytochrome c oxidase I [mtCOI]. For the 18S rRNA gene, three regions were amplified: 18S-1F1/18S-1R632, 18S-2F576/18S-2R1209, and 18S-3F1129/18S-R1172^74^. For mtCOI, we amplified one region: Dorid_COI.3 F/Dorid_COI.1R^73^. Primer sequences are presented in Table 5. The expected length of the fragments was between 680 and 780 bp. We used polymerase chain reaction (PCR) to amplify DNA using a C1000 Touch (Bio-Rad, Hercules, CA) thermocycler. PCR reactions consisted of 2.5 µM of each primer, 2.0 µl of template DNA, 5 µl of 2X PCR buffer (Phusion^®^ Hot Start Flex, Thermo Scientific, Foster City, CA), and 0.5 µl MgSO_4_ in a 10-µl reaction. 18S rRNA was PCR-amplified with an initial activation step of three minutes at 98 °C, followed by 35 cycles of denaturation (30 seconds at 98 °C), annealing (30 seconds at 54 °C), and extension (30 seconds at 72 °C) with a final extension step (10 minutes at 72 °C). Only the first of the three regions for 18S rRNA (18S-1F1/18S-1R632) was used for analysis because the other two did not amplify consistently. mtCOI was PCR-amplified with an initial activation step of 98 °C, followed by 30 cycles of: denaturation (30 seconds at 98 °C), annealing (30 seconds at 45 °C), and extension (60 seconds at 72 °C) with a final step of five minutes at 72 °C. The size of the PCR amplicons was checked in a 1.5% agarose gel. PCR products were sequenced in both directions using the amplification primers at Molecular Cloning Laboratories (San Francisco, CA).

**Table 5:** PCR and sequencing primers of 18S and COI genes used in this study.

Primer name	Sequence 5′-3′	Length	Reference
18S - 1F1	AACCTGGTTGATYCTGCCAG	1780 bp	Nishitani *et al*. 2012
18S - 1R632	ACTACGAGCTTTTTAACYGCARC	1780 bp	Nishitani *et al*. 2012
Dorid COI - 3 F	AAGGWATACCTACAGAAAARATACC	684 bp	Williams *et al*. 2017
Dorid COI - 1 R	CTGTGAATAGRGGRAATCAGTTTAT	684 bp	Williams *et al*. 2017

### Molecular analysis

We combined forward and reverse complementary sequences of 18S rRNA and mtCOI genes using Geneious (version 11.0.5) to create consensus sequences. The consensus sequences were submitted to NCBI and registered in GenBank (accession nos. in Table 1 and Supplementary Table 1). The 18S rRNA sequences we generated with primers 18S-1F1 and 18S-1R632 were approximately 660 bp in length, but were trimmed the final alignment to a common length of 614 bp to remove poorly aligned terminal ends. Similarly, mtCOI sequences were initially 680 bp in length and were trimmed to 554 bp for analysis. Initially, we aligned our partial consensus sequences of 18S rRNA and mtCOI genes with sequences from the *Polydora websteri* and other species in the genus *Polydora*, obtained from GenBank (Table 6). For this alignment we only employed Genbank sequences that have been published along with a clear morphological description of the species. We reconstructed phylogenetic trees using the neighbor-joining method based on Kimura 2-parameter model with 1000 bootstrap replications. We used a gamma distribution (shape parameter = 1) to mode the rate variation among sites. The 18S rRNA analysis involved 25 nucleotide sequences. Codon positions included were 1st+2nd+3rd+Noncoding. All positions containing gaps and missing data were eliminated. There were a total of 566 positions in the final dataset and the optimal total branch length was 0.097. The mtCO1 analysis involved 58 sequences and a total of 540 positions with an optimal total branch length of 1.2. We used the Molecular Evolutionary Genetics Analysis software (MEGA version 7.0.26), with *Pseudopolydora dayii Simon* as an outgroup. We used MEGA 7.0.26 to determine the haplotype diversity, nucleotide diversity and the average number of nucleotide differences (Table 2). Pairwise distance for intraspecific and interspecific polydorid species for mt COI (six species, Table 3) and nuclear 18S (three species, Table 4), were also calculated using MEGA 7.0.26 with Kimura’s two-parameter method with a gamma rate variation distribution. The Kimura two-parameter metric was chosen to facilitate comparison with previous studies^52^. The sequences used to calculate these distances were retrieved from GenBank and from our own dataset (Tables 3 and 4).

**Table 6:** Details for 18S and COI sequences from GenBank that were used for phylogenetic analyses.

Species	Acc No.	Country	Host	Ref.
**Gene 18S rRNA**				
*Polydora websteri*	AB705402	Japan	*Crassostrea gigas*	^63^
*Polydora hoplura*	AB705410	Australia	*Haliotis laevigata*	^63^
*Polydora aura*	AB705409	Japan	*Crassostrea gigas*	^63^
*Polydora onagawaensis*	AB691768	Japan	*Crassostrea gigas*	^75^
*Polydora calcarea*	AB705403	Japan	*Crassostrea gigas*	^63^
*Polydora brevipalpa*	AB705407	Japan	*Mizuhopecten yessoensis*	^63^
*Polydora lingshuiensis*	KF562238	China	*Pinctada imbricata*	^76^
*Polydora nuchalis*	KY677902	South Africa	N/A	^73^
*Pseudopolydora dayii*	KY677907	South Africa	N/A	^73^
**Gene mtCO1**				
*Polydora websteri*	MG9777402 to MG9777414	United States	*Crassostrea virginica*	^10^
*Polydora websteri*	KR337462, KR337464, KR337466, KR337467, KR337468, KR337472	China	*Crassostrea gigas*	^52^
*Polydora brevipalpa*	KR052122 to KR052199 & KP231319	China	*Mizuhopecten yessoensis*	^77^
*Polydora cornuta*	EF525280 to EF525282 & EF525289	Various	N/A	^72^
*Polydora aura*	KR052136 to KR052140	China	*Anadara uropigimelana*	^52^
*Polydora hoplura*	KY677861 to KY677865 & KY677910	South Africa	N/A	^73^
*Polydora nuchalis*	KY677866	South Africa	N/A	^73^
*Pseudopolydora dayii*	KY677868	South Africa	N/A	^73^

## Supplementary information

Supplementary Dataset 1.

Supplementary Table 1.
