# Supplementary material for: Confirmation of the shell-boring oyster parasite Polydora websteri (Polychaeta: Spionidae) in Washington State, USA
Source: Sci Rep. 2020 Mar 3;10:3961. doi: 10.1038/s41598-020-60805-w (PMC7054429; doi:10.1038/s41598-020-60805-w)
Supplement: Supplementary file 2 — Supplementary Table 1. [file 41598_2020_60805_MOESM2_ESM.docx]

**SUPPLEMENTARY TABLE**

**Confirmation of the shell-boring oyster parasite *Polydora websteri* (Polychaeta: Spionidae) in Washington State, USA**

Julieta C. Martinelli^1*^, Heather M. Lopes^1^, Lorenz Hauser^1^, Isadora Jimenez-Hidalgo^1^, Teri L. King^2^, Jacqueline L. Padilla-Gamiño^1^, Paul Rawson^3^, Laura H. Spencer^1^, Jason D. Williams^4^, Chelsea L. Wood^1^

**Table 1.** Taxa, sampling location data, museum catalog numbers of voucher specimens and GenBank accession numbers of specimens for which we have either molecular or morphological identifications. The rest of the specimens that have both molecular and morphological identification are presented in Table 1. USNM = National Museum of Natural History, Smithsonian Institution, Washington D.C., USA; SEM = specimen prepared for scanning electron micrograph; EtOH = specimen preserved in ethanol.

| **Molecular ID** | **Morphological ID** | **Worm ID on trees** | **Location and host** | **Coords.** | **Date** | **Museum Voucher (SEM or EtOH)** | **GenBank Accession Numbers** | |
| --- | --- | --- | --- | --- | --- | --- | --- | --- |
|  |  |  |  |  |  |  | ***18S*** | ***COI*** |
| *Polydora websteri* | *Polydora websteri* | - | Oakland Bay, Washington State, USA; from shells of *Crassostrea gigas* | 47° 13' 45.93", –123° 3' 19.43 | 15 Aug 2018 | H1-61-1(SEM) | MK695999 | - |
| *Polydora websteri* | *Polydora websteri* | - |  |  |  | H1-61-3(EtOH) | - | - |
| *Polydora websteri* | *Polydora websteri* | - |  |  |  | H1-61-4(SEM) | - | - |
| *Polydora websteri* | *Polydora websteri* | - |  |  |  | H1-61-5(EtOH) | MK696002 | - |
| *Polydora websteri* | *Polydora websteri* | OAK11 |  |  |  | H1-61-6(SEM) | MK696000 | MK696586 |
| *Polydora websteri* | *Polydora websteri* | OAK12 |  |  |  | H1-61-7(SEM) | MK696001 | MK696587 |
| *Polydora websteri* | *Polydora websteri* | OAK12 |  |  |  | H1-61-8(SEM) | MK696003 | MK696588 |
| *Polydora websteri* | *Polydora websteri* | - |  |  |  | H1-61-9(SEM) | - | - |
| Unident. polydorin | - | - | Totten Inlet, Washington State, USA; from shells of *Crassostrea gigas* | 47° 9' 43.09",  –122° 59' 19.62" | 18 Sep 2017 | - | MH891524 | MK188738 |
| Unident. polydorin | - | - | Same as above | Same as above |  | - | MH891525 | MK188739 |
| Unident. polydorin | - | - |  |  |  | - | MH891527 | MK188740 |
| Unident. polydorin | - | - |  |  |  | - | MH891530 | - |
| Unident. polydorin | - | - |  |  |  | - | MH891528 | - |
| Unident. polydorin | - | - |  |  |  | - | MH891536 | - |
| Unident. polydorin | - | - |  |  |  | - | MH891534 | - |
| Unident. polydorin | - | - |  |  |  | - | MH891531 | - |
| Unident. polydorin | - | - |  |  |  | - | MH891532 | - |
| Unident. polydorin | - | - |  |  |  | - | MH891523 | - |
| Unident. polydorin | - | - |  |  |  | - | MH891535 | - |
| Unident. polydorin | - | - |  |  |  | - | MH891533 | MK188741 |
| Unident. polydorin | - | - |  |  |  | - | MH891529 | MK188742 |
| Unident. polydorin | - | - |  |  |  | - | MH891526 | MK188743 |
| *Polydora websteri* | *Polydora websteri* | LI1 | North Sea Harbor, Long Island, New York, USA; from shells of *Crassostrea virginica* | 40° 56' 24.13"N, 72° 25' 3.97"W | 12 Sep 2018 | P1-109-2a, b | MK369933 | MK696582 |
| *Polydora websteri* | *Polydora websteri* | LI2 | Same as above | Same as above |  | P1-109-3a, b | MK369934 | MK696583 |
| *Polydora websteri* | *Polydora websteri* | LI3 | Same as above |  |  | P1-109-4a, b | MK369935 | MK696584 |
| *Polydora websteri* | *Polydora websteri* | LI 4 | Same as above |  |  | P1-109-5a, b | MK369936 | MK696585 |
